# A Systems Approach to Homeostasis: What Euryhaline Fish Teach Us About Organismal Stress Responses

**DOI:** 10.1093/icb/icaf085

**Published:** 2025-06-24

**Authors:** Alexander A Mauro, Jonathan P Velotta, Cameron K Ghalambor

**Affiliations:** Department of Biology, Norwegian University of Science and Technology (NTNU), N-7491 Trondheim, Norway; Department of Biological Sciences, University of Denver, Colorado, 80210 , USA; Department of Biology, Norwegian University of Science and Technology (NTNU), N-7491 Trondheim, Norway

## Abstract

Historically, organismal biologists have studied the organism’s response to environmental variation from two complementary perspectives: one has focused on “stability” and the capacity of organisms to maintain a constant internal state (e.g., homeostasis) across environments, whereas the other has focused on “change” and how the expression of traits varies as a function of a continuous environmental factor (e.g., performance curves). While these approaches differ, they rely on the same fundamental principles dispersed across cell biology, physiology, endocrinology, ecology, and evolution and thus could be better integrated. Through the lens of systems biology, we offer a perspective that explores the idea that organisms maintain stability of critical physiological functions during environmental change through changes of lower-level traits within physiological regulatory networks. We assert that such network thinking and an emphasis on the cost of homeostatic systems are critical when relating the physiological responses of cells, tissues, hormones, etc to whole-organism performance and the ecological context in which the responses occur. We suggest that such an approach has the potential of transcending levels of biological organization by connecting approaches typically studied in isolation of each other and that this will help the organismal biologist relate physiological responses measured in the lab to performance and fitness in natural settings. To illustrate our perspective and aid in our presentation of practical tips for the experimental biologist, we use examples from our own research on osmoregulation in euryhaline fish.

## Introduction

Organismal biology is a multidisciplinary field united by the guiding principle that the organism is a complex, integrated entity that is ultimately embedded within its natural environment. From this perspective, insight can be gained by examining different levels of biological organization (i.e., gene, cell, organism) separately, but the best insight stems from integrating these studies and placing them within the context of larger themes, like how organisms respond to environmental change (Schwenk et al. 2009; Mykles et al. 2010). Historically, organismal biologists have studied responses to environmental change from two complementary perspectives. One approach focuses on “stability,” or the capacity of organisms to maintain a constant internal state across a range of environments (Pennazio 2009; Woods and Wilson 2015), such as internal temperature in endotherms (e.g., Cossins and Bowler 1987) or internal osmolality in euryhaline fish (Schultz and McCormick 2012). The mechanisms underlying such stability involve integrated cellular, developmental, physiological, and behavioral changes and are often studied in the context of understanding when an organism enters a “stressful state” (McCormick 2009; Wingfield 2013; Kültz 2020). A complementary approach focuses on “change” and considers how the expression of a trait varies as a function of an environmental factor (Little and Seebacher 2021). The most familiar use of this approach is the thermal performance curve, which measures organismal performance (i.e., escape speed, fecundity, etc) as a function of temperature, but such curves can be developed for a variety of traits and environmental factors (Huey and Stevenson 1979; Huey and Kingsolver 1989; Sinclair et al. 2016). The motivation behind studying performance curves is that they represent how environmental factors influence the lower-level biophysical, cellular, and physiological functions underlying whole organismal responses (Huey and Stevenson 1979; Huey and Kingsolver 1989). While the approaches used to study stability and change in response to environmental variation are often employed separately and emphasize different physiological mechanisms, they are connected by shared principles stemming from cell biology, physiology, endocrinology, ecology, and evolution (Schwenk et al. 2009; Padilla and Tsukimura 2014). Hence, we suggest these perspectives can be better united and propose that systems biology (Mykles et al. 2010; Giudice et al. 2018; Bogdan et al. 2021) holds potential to provide a common conceptual framework.

Systems biology in its broadest sense is the application of principles and theories in which the processes and interactions between different biological components of a system are emphasized over any one component itself (Kirschner 2005; O'Malley and Dupré 2005; Soyer and O'Malley 2013). Emergent features of the “systems approach” to biology include an emphasis on the network structure to biological phenomena (i.e., gene networks, food webs, physiological regulatory networks), the hierarchical structure of biological phenomena (i.e., cells, organisms, communities, ecosystems), and the processes and entities that link parts of a system (hormones, integrator molecules, energy, nutrients, information) (Kirschner 2005; Jorgensen et al. 2007; Wagner et al. 2007; Crombach and Hogeweg 2008; Cohen et al. 2012; Soyer and O'Malley 2013; Woods and Wilson 2013; Martin and Cohen 2015; Fischer et al. 2016). Systems biology has been applied extensively to cell and gene regulatory networks, developmental processes, and whole ecosystem processes like food webs and nutrient cycling (Purdy et al. 2010; Soyer 2012; Green et al. 2015; Huang 2018). However, recently there has been an increased interest in—and application of—systems approaches to organismal biology that focus on several overarching themes, including stress, homeostasis, plasticity, and adaptive evolution (Padilla and Tsukimura 2014; Giudice et al. 2018; Velotta and Cheviron 2018; Schwartz 2020).

Here, through the lens of systems biology, we offer a perspective that explores the idea that organisms maintain “stability” of critical physiological functions in the face of environmental variation through the “change” of lower-level traits within the same physiological regulatory network(s). We assert that such network thinking and an emphasis on the cost of homeostatic systems are critical when relating the physiological responses of cells, tissues, hormones, etc to whole-organism performance and that this will help the organismal biologist relate physiological responses measured in the lab to performance in natural settings. Concepts from homeostasis, stress, and systems biology are explored, but these fields are not reviewed as such reviews already exist (e.g., Romero et al. 2009; McEwen and Wingfield 2010; Sokolova 2013; Schulte 2014; Del Giudice et al. 2018). Instead, we provide examples from our research on osmoregulation in euryhaline fish to make our ideas less abstract and illustrate how our ideas can be applied. We end with suggestions and practical tips for experimental biologists interested in applying an “organismal systems” approach to their own research.

## Integrating organismal biology and systems biology

### Homeostasis, physiological regulatory networks, and homeostatic costs

In this section, we introduce key concepts that link systems biology and organismal biology. Vital to both fields is the idea that for a system to function properly under diverse circumstances, some variables must change so others are stable (McEwen and Wingfield 2010; Schulte et al. 2011; Woods and Wilson 2015; Nijhout et al. 2017). In systems biology this is conceptualized as “robustness balanced by plasticity” with robustness being “The ability of a biological system to resist otherwise deleterious genetic or environmental variation” (Nijhout et al. 2019) and plasticity being the ability of the same genotype/individual to produce different phenotypes under different environments (West-Eberherd 2003). For example, robustness in developmental pathways often leads to the proper development of the organism despite genetic mutations or environmental influences because of redundancy, plasticity, and the capacity for compensatory changes within the genotype to phenotype map (Dalziel et al. 2009; Fischer et al. 2016; Nijhout et al. 2019). Similarly, in physiology, parameters essential to whole organism function, such as pH, internal temperature, plasma osmolality, glucose levels, and calcium levels often remain within vital bounds despite environmental change (Cannon 1929) because of compensatory changes in an organism’s physiological regulatory network (see Box 1 and below) (Cohen et al. 2012; Martin and Cohen 2015). It is debated whether the term homeostasis fully encompasses this concept of stability in parameters through compensatory changes, as various alternative terms or models have been proposed (see Romero et al. 2009; McEwen and Wingfield 2010; Woods and Wilson 2015). For our purposes here, we define *homeostasis* as “the process by which biological control systems regulate essential physiological factors within some bounds” (Cannon 1929; Woods and Wilson 2015) and argue that it is the organismal counterpart to system’s robustness.

**Box 1: What is a physiological regulatory network?**

The term *Physiological Regulatory Network* or *PRN* was first introduced by Cohen et al. (2012) as “the network of molecules and their regulatory relationships that maintain and adjust homeostasis and facilitate performance at the whole-organism level.” Below we discuss several key aspects of the PRN.

**Ultimately, each organism can be defined as a single system with a single PRN**. As originally defined, each individual is a single system with a single *PRN structure*. An individual’s PRN structure is determined by genetic and developmental variation; hence, individuals within a species have similar but not identical PRN structures (Martin and Cohen 2015). Environmental variation causes PRN structures to take on different states or configurations, which define the whole organism response to the environment.

**The nodes and edges of a PRN change depending on the scope of the study**. Most broadly, PRN nodes are defined as “circulating molecules or their functional complexes” and edges as “co-regulatory patterns of nodes” (Martin and Cohen 2015). Hence, a hormone, receptor, or cell can be a node. However, an entire complex of molecules, like the receptors, gene products, cells, etc. within a tissue or organ, can also be a node. If organs are nodes, then the integrator molecules responsible for their co-regulatory patterns are the edges. Therefore, hormones, which are ubiquitous integrator molecules in regulatory networks, could be considered a node or an edge (Martin et al. 2011). This highlights the hierarchical nature of the PRN: PRNs contain many sub-networks or *sub-PRNs* that operate in semi-isolation from one another, but because the sub-PRNs are ultimately part of the same PRN, they are not in complete isolation.

**The function of a PRN is defined by whole-organism performance**. Because each organism is a single system containing a single PRN made up of many sub-PRNs, the “performance” of the PRN is equivalent to whole-organism performance, which is in turn conditioned on the environment in which performance is measured. Further, the environment will dictate what state the PRN is in (Martin and Cohen 2015). Thus, a PRN becomes less meaningful if it is not in the context of the whole organism and its environment.

**The PRN is (for now) a hypothesis for how the organism responds to change**. Presently, measuring a whole organism PRN is an unrealized goal due to both gaps in measurement capability and in theory that can properly integrate the required data (Martin and Cohen 2015). However, it is possible to conduct experiments that provide evidence for how sub-PRNs operate, how sub-PRNs relate to one another, and how this all relates to organism performance (see Box 2). Hence, researchers can make informed hypotheses about the configuration of the PRN as it responds to environmental variation, and this is a key step in understanding the mechanistic basis for how organisms respond to changes in their environments.

**Box 2: Practical insights for the experimental biologist**

Below we outline key questions that will assist experimental biologists designing studies in light of the concepts presented. We also suggest types of measurements/experiments that could help with answering each question. However, we note that the answers to these questions will be highly study system dependent and that great insight is gained by answering only some of the questions below.

**Figure:**
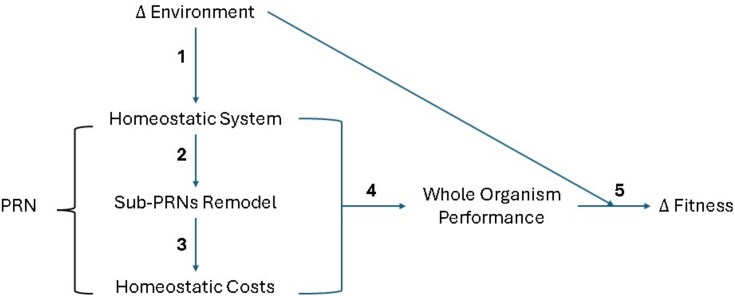


**A schematic depicting the connections that relate to the questions below**

1. What homeostatic parameter (blood pH, osmolality, internal temperature, calcium levels, etc) is the environmental change threatening? This can often be measured directly in the lab or intuited from studies on model organisms.
2. How do sub-PRNs respond to the environmental change? Investigating this question will depend on the scope of the study and a priori knowledge of the sub-PRNs. Potential methods include multi/single tissue transcriptomics, proteomics, or hormone assays.
3. How does the change in sub-PRNs lead to homeostatic costs? Potential methods include changes in metabolic rate/aerobic scope, assays of chemical markers of stress, and assays of traits related to potential tradeoffs with the homeostatic response.
4. How does the response of the homeostatic system lead to a change in whole-organism performance? Potential performance metrics include growth rate, body condition change, survival, reproduction rate, locomotor performance, etc.
5. How does a change in performance impact fitness in a natural or natural-like environment? Many of the whole-organism performance metrics listed above are common fitness proxies and can be used to measure fitness, but it should be emphasized that fitness is best measured in natural or natural-like environments.

The mechanisms regulating homeostasis in the “organismal system” have been described as an organism’s *physiological regulatory network* (PRN). A PRN is the full suite of molecules, cells, and tissues and their regulatory relationships that mediate homeostasis and organismal performance (for the original description, see Cohen et al. 2012; Martin and Cohen 2015). While the PRN is conceptually appealing because it considers the organism as a single system that contains one network governing all physiological processes, studying all the regulatory relationships within a whole organism is not feasible. Instead, empirical research is largely focused on the sub-networks or *sub-PRNs* which make up the larger, organism-wide network. For example, the nodes of a sub-PRN can be organs, and the edges can be the ways in which the organs communicate (i.e., via signaling molecules in the neuroendocrine system). However, each individual organ could also be considered a system containing one or more sub-PRNs in which tissues or cells are nodes. The key point is that each sub-PRN is part of—and interacts with—the organism-wide PRN, which shifts the perspective from focusing on any single mechanism in isolation to one that explicitly recognizes connections between levels of biological organization and seeks to understand how any given mechanism or response is imbedded within a larger set of networks. Although our understanding of biological networks is increasing (Martin and Cohen 2015; Bogdan et al. 2021), in most cases researchers will need to generate and test alternative hypotheses for what defines the nodes and edges of their system (see Box 1). Nevertheless, systems thinking, particularly the PRN, can improve our understanding of how physiological processes govern organismal performance (Martin and Cohen 2015).

Similarly, network thinking can aid in our understanding of the costs of homeostatic systems (Romero et al. 2009; Del Giudice et al. 2018). The costs to homeostasis were first outlined explicitly via the concept of allostasis (Sterling and Eyer 1988) and termed “allostatic loads” (McEwen and Wingfield 2003, 2010). Allostatic loads come in two types: Type 1 includes the energetic cost of a homeostatic system, and Type 2 includes the pathologies associated with prolonged overactivation of a homeostatic system (Romero et al. 2009; McEwen and Wingfield 2010). Trade-offs between organismal functions that are controlled by the same sub-networks represent one mechanism by which type 2 costs occur (Welch and Waxman 2003; Wagner et al. 2008; Mauro et al. 2020). For example, hormones, which often serve as signaling molecules for multiple systems, may be excreted to stabilize one system but simultaneously decrease performance in a related system (i.e., hormonal pleiotropy; Cox et al. 2016; Dantzer and Swanson 2017). To avoid confusion with prior uses of “allostatic load” and to remain as general as possible while including network concepts, we will use the term “*homeostatic costs*” when discussing the costs of a homeostatic system (for a discussion of terms relating to homeostasis and allostasis, see Romero et al. 2009; McEwen and Wingfield 2010; Woods and Wilson 2015).

### Ecological consequences of homeostatic systems responding to environmental stress

Here, we use the concepts discussed above to highlight how systems thinking can assist in the organismal biologist’s aim to place physiological responses to environmental variation in an ecological context. We begin by incorporating concepts from stress biology and define *stress* as occurring in situations in which environmental demand challenges homeostasis enough to elicit a response that incurs a non-zero fitness cost (Koolhaas et al. 2011; Schulte 2014; Giudice et al. 2018). The relationship between stress and homeostasis is well outlined in two integrative models: the “Reactive Scope Model” (Romero et al. 2009) and the “Energy-Limited Tolerance to Stress Framework” (Sokolova et al. 2012; Sokolova 2013), which collectively describe the homeostatic costs of a system at four different stress states: (1) an “optimal” state in which homeostasis is easily achieved, the organism has excess energy to spend on tasks linked to fitness, and fitness is high, (2) a sub-optimal “pejus” state in which excess energy is generally absent, maintaining homeostasis incurs costs, but the individual may be able to persist (i.e., grow, reproduce, survive), (3) a sub-sub-optimal “pessium” state in which the costs to maintain homeostasis are not sustainable in the long term and the organism is in an energy deficit state, but the system is preventing whole-organism failure, and (4) a final “lethal” state in which homeostasis of vital functions is lost and short term persistence is not possible. Below we use the terms optimal, pejus, pessium, and lethal to refer to the organismal stress states (see Fig. 2).

An emergent point from integrating concepts from stress biology into our discussion of systems biology and organismal biology is that the duration of the stressor influences how sub-PRNs remodel themselves. After an environmental stressor is detected, an acute stress response is mounted within minutes to hours (i.e., acute stress) (Romero et al. 2009; Gormally and Romero 2020), which leads to “short-term” remodeling of sub-PRNs meant to quickly stabilize the system. However, if the stress persists (or is anticipated to persist, i.e., chronic stress), the system may adopt a chronic response with a “long-term” remodeling of the sub-PRNs, which may reduce some of the homeostatic costs. For example, cold hardening in insects, the process by which insects remodel their physiology to withstand cold temperatures, can happen in minutes (rapid hardening) in response to sudden cold snaps or over the course of days to weeks in response to cooling temperatures and shortening photoperiod (seasonal/acclimatory hardening) (Sinclair et al. 2003; Teets and Denlinger 2013). However, the physiological mechanisms used in the different forms of cold hardening are somewhat distinct. Rapid cold hardening is largely controlled via calcium signaling and features an inhibition of apoptotic cell death because the baseline response would be a costly overactivation of cell death. In contrast, seasonal cold hardening is not known to interact with apoptotic cell death (and hence regular cell regulation occurs) but uniquely features changes in antifreeze protein abundance that are controlled via changes in gene expression (Teets and Denlinger 2013). Hence, an “optimal” remodel or plastic change of sub-PRNs in response to an environmental shift depends to some degree on the expected duration and frequency of the stress (McNamara and Buchanan 2005; Romero et al. 2009).

This same logic applies to a short-term plastic ecological response versus an evolutionary change of the PRN if populations experience an environmental shift for generations. Here, there should be selection to alter the PRN so that homeostatic functions are maintained while homeostatic costs are minimized. For example, there is evidence that organ size evolves in response to heat stress to lower the relative metabolic cost of the organ(s) involved in temperature regulation (Russell and Chappell 2007; Wiersma et al. 2012; Careau et al. 2015). There is also evidence that changes to receptors or other downstream elements in networks—as opposed to alterations further upstream—are evolutionarily favored because it reduces the likelihood the change leads to tradeoffs with other functions (a homeostatic cost) (Sommer and Mayer 2015; Mauro et al. 2020). For example, Di Poi et al. (2016) found changes in cortisol receptors but not upstream elements of the glucocorticoid axis in a stickleback population that had been adapting to freshwater for multiple generations and posited this could allow the population to maintain the osmoregulatory function of cortisol without potentially introducing pleiotropic effects of cortisol. Hence, the homeostatic costs of the “evolved” freshwater PRN were reduced compared to the plastic “remodeled” PRN of the ancestral marine phenotype when measured in freshwater.

**Fig. 1:**
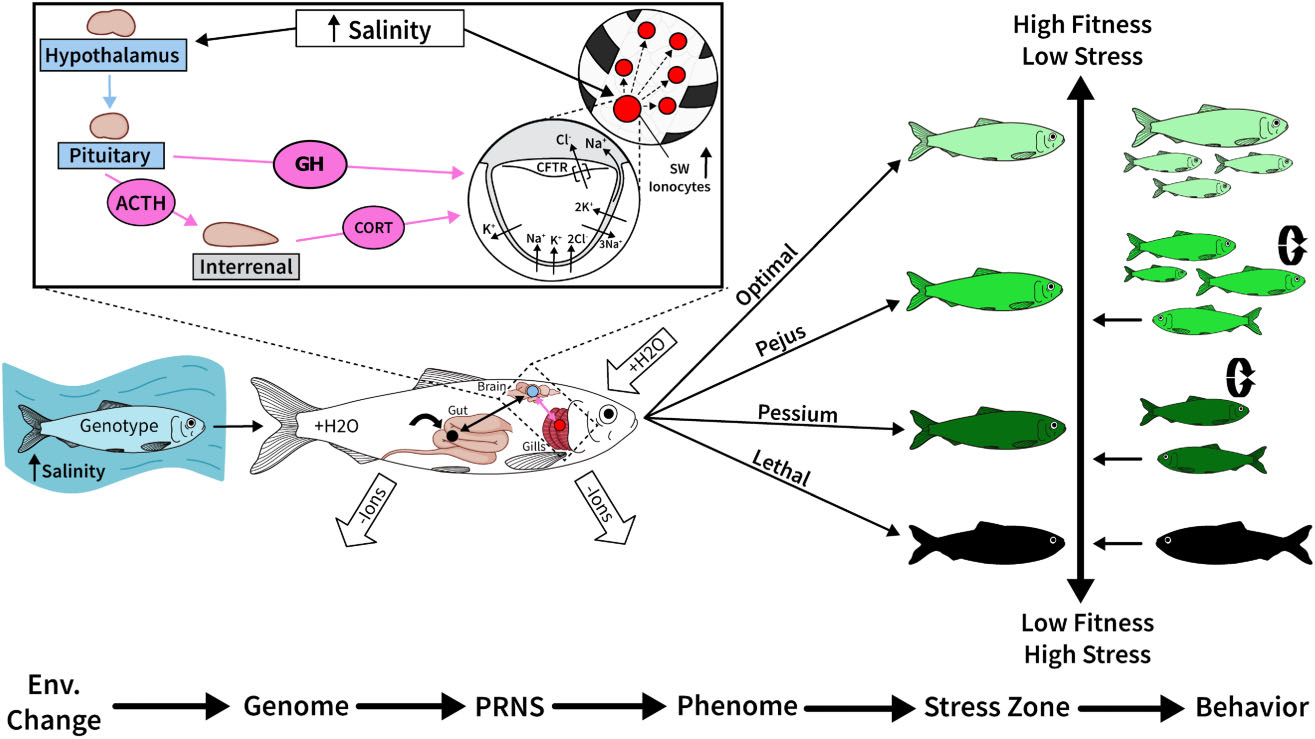
When external salinity increases, euryhaline fish try to maintain constant internal osmotic pressures (homeostasis) by altering their osmoregulatory sub-PRN to intake more water and excrete excess ions. Above is a schematic highlighting some (not all) of this homeostatic response. Osmosensors in the gut, gills, and brain detect internal changes in osmotic pressure, which leads to a endocrine response. Highlighted here are two hormonal responses stemming from the Hypothalamus-Pituitary-Interrenal axis: (1) ACTH released from the pituitary stimulates the release of cortisol (CORT) from the interrenal into the bloodstream, and (2) GH is released into the bloodstream from the pituitary. CORT and GH then bind to receptors in the gill, which triggers the hypo-osmoregulatory function of the gills: the proliferation of saltwater type ionocytes. These ionocytes then sense osmotic changes and excrete excess ions via ion transporters (see text for specifics). Hence, although the gill, brain, and gut form their own trans-organ sub-PRN, each organ itself also contains a smaller sub-PRN, and communication between the tissues is facilitated by the neuroendocrine system (connection between the brain and gill). Depending on the severity of the (salinity) stress and the makeup of the individual’s sub-PRNs, which is determined by the individual’s genotype to phenotype map, the fish will enter one of 4 zones of stress: Optimal, Pejus, Pessium, Lethal. These zones are described in detail in Fig. 2, and the behavioral consequences are highlighted here. Depending on the zone, an individual will either engage in: fitness increasing tasks like reproduction (baby fish), behavioral regulation to reduce stress (circular arrows), or fleeing the environment (left pointing fish). Artwork by Emily Tarnawa.

**Fig. 2:**
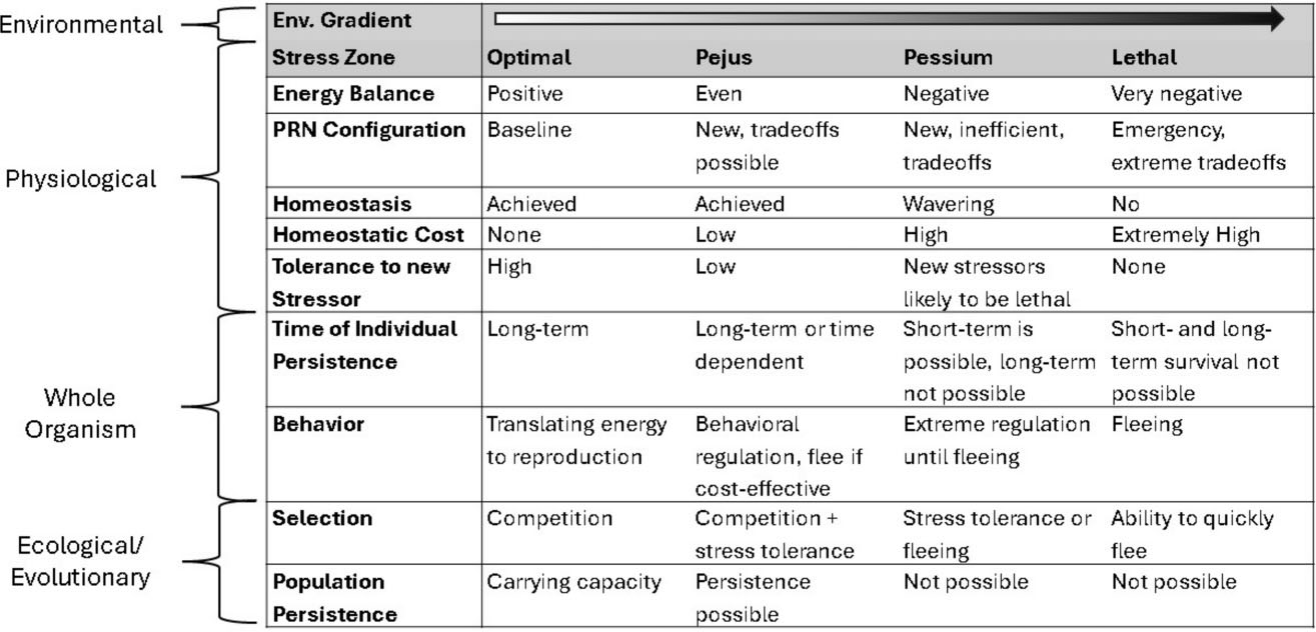
An abiotic stressor that corresponds to a continuous environmental factor will cause an organism to enter one of four stress states, Optimal, Pejus, Pessium, or Lethal, as the organism experiences change in that environmental factor. The physiological, whole organism, and ecological/evolutionary consequences of an individual or population residing in each of these four zones are summarized here and discussed in the main text.

Lastly, the homeostatic cost of different stress states influences the behavioral/physiological strategy an organism may take to navigate the stressor and the selective pressures that organism may face due to the stressor. If an environmental shift increases the homeostatic costs of persisting in that environment, an organism has three options: (1) remain in the environment and accumulate costs, which may reduce over time as its PRN is remodeled via plasticity/acclimation, (2) spend energy/incur a cost to behaviorally regulate to reduce exposure to the stressor, or (3) spend energy/incur a cost to disperse far enough so the environmental stressor is absent. The optimal strategy is the one that achieves the highest fitness as a function of the homeostatic costs and opportunity costs (access to mates, food, etc) of the available options (McNamara and Buchanan 2005; Romero et al. 2009; Bonte et al. 2012; Sokolova 2013). Hence, behavioral avoidance of poor environments or dispersion to higher quality environments should be favored if the overall cost of the behavior/dispersal is less than the cost of staying (Bonte et al. 2012). Thus, as the environment becomes more stressful, selection will favor the ability to detect and disperse away from the stressor over the ability to stay and tolerate it (Fig. 2; Duckworth 2009; Romero et al. 2009; Schulte 2014). Therefore, one would predict that organisms would switch from “weathering” a costly environment to fleeing it somewhere between the pejus and pessium zones (Sokolova 2013) (Figs. 1 and 2), and this would correspond to the organism’s distribution in nature (Holt and Keitt 2005; Sexton et al. 2009). For instance, the distributions of many marine organisms are predicted to contract due to changes in temperature and oxygen content, which will make parts of their current distributions too energetically costly (Deutsch et al. 2015). This perspective suggests that mechanisms that enable organisms to detect oncoming stressful environmental shifts and anticipatorily remodel their PRN should be favored if the maintenance/use of such systems has a lower cost than experiencing the shift. For example, changes in photoperiod during the spring trigger changes to sub-PRNs in many woody plants, some of which aid in transitioning and tolerating the forthcoming seasonal temperature increase (e.g., Ettinger et al. 2021). In those cases where predictable environmental shifts occur (e.g., seasonal shifts), anticipatory remodeling of sub-PRNs can be a low-cost homeostatic response (see Romero et al. 2009).

## Osmoregulation in euryhaline fish as a homeostatic system

In this final section, we use euryhaline fish’s ability to maintain a stable internal osmolality despite fluctuations in external salinity to demonstrate how the concepts discussed above can be applied in a natural system. We first provide background on euryhalinity through the lens of systems biology and then discuss two case studies: the Trinidadian guppy in lowland estuaries and migratory Alewives that have become landlocked in lakes. While this section is specific to osmoregulation, we suggest the lessons can be applied more broadly across any homeostatic system and discuss this in the conclusion and Box 2.

### A systems perspective on osmoregulation in euryhaline fish

Euryhalinity is the ability to tolerate both freshwater (<0.5 ppt salt) and seawater (∼35 ppt) and, although relatively rare, is widespread across the teleost fish phylogeny (Schultz and McCormick 2013). Teleost fish maintain an internal osmotic pressure of approximately 1/3^rd^ seawater (∼12 ppt); thus, fish in freshwater are at a higher internal osmotic pressure than their environment, where as fish in seawater are at a lower internal osmotic pressure than their environment. Therefore, when a euryhaline fish moves into saltwater (<0.5–2 ppt) from freshwater, it must switch from performing *hyper*-osmoregulation to *hypo*-osmoregulation to maintain a constant internal osmolality and hence osmotic/ionic homeostasis (Edwards and Marshall 2012). Broadly, this switch requires that euryhaline fish increase (water) drinking and salt excretion so that excess water is retained in the extracellular fluid while the salts enter the bloodstream and are excreted at the gills (Baldisserotto et al. 2007). However, this requires a highly integrated response across diverse systems, as (1) the change in salinity must be sensed, (2) that information must be processed, transformed, and communicated across the whole organism, and (3) the organs associated with osmoregulation (brain, gills, gut, and kidney) must mount a coordinated response across sub-PRNs to retain more water and excrete excess ions.

In response to stress induced by changes in extra- and intra-cellular osmotic pressure, fish use a “combinatorial” set of osmotic sensors and signal transduction pathways to finely regulate their osmoregulatory response to restore homeostasis ([Bibr bib44][Bibr bib45]). Osmosensing involves several mechanisms, including baroreception of systemic circulation at the carotid and aortic arches, direct sensing of changes in ion strength, macromolecular density, and cytoskeletal strain that results from changes in osmosis in the epithelial cells of osmoregulatory organs such as the gills and gut, and the endocrine system responding to signals originating in the brain and pituitary gland (Kultz 2012). Once a change in internal osmotic pressure is sensed, a multitude of electrical, endocrine, paracrine, and autocrine signals are released that stimulate a series of effector mechanisms at osmoregulatory organs to mediate adjustments of osmotic pressure back to homeostasis. While there have been considerable advances in our understanding of these mechanisms, few studies have considered osmoregulation from a systems perspective.

In a PRN systems context, we can consider cells within osmoregulatory tissues that sense changes in osmotic pressure—and those that respond to it—to be nodes within a sub-PRN, while the edges are the electrical, endocrine, and paracrine/autocrine signaling molecules that transduce osmotic sensations. At a higher organizational level, osmoregulatory organs can themselves be considered nodes of a sub-PRN in which hormones are the edges that connect them (McCormick 2001; McCormick and Bradshaw 2006). For example, cortisol—whose secretion occurs at the head kidney (interrenal) and is controlled via a hormonal cascade beginning in the hypothalamus and pituitary—binds to glucocorticoid receptors in the gills and stimulates the production of a highly specialized ion secretory cell called an ionocyte (Fig. 1; Rotllant et al. 2000; McCormick and Bradshaw 2006; Hwang et al. 2011; Edwards and Marshall 2012). Combined with growth hormone (GH), cortisol also stimulates the upregulation of an integrated set of well-characterized ion pumps and transport proteins in gill ionocytes, including Na+/K+−ATPase and Na+/K+/2Cl− co-transporter, as well as a suite of junction proteins called claudins, all of which increase Na + and Cl− excretion across ionocytes and through the “leaky” tight junctions in between ionocytes and accessory cells (Takei and McCormick 2013 ). Hence, the gill, which contains its own sub-PRN, is remodeled at the molecular level after detected changes in osmotic pressure through the differentiation of cell types and differential regulation of gene products, which are stimulated by cortisol—whose secretion is controlled by other organs with their own sub-PRNs (Fig. 1). Similar changes and processes also occur in other organs during osmoregulation (for reviews, see McCormick 2001; Baldisserotto et al. 2007; Edwards and Marshall 2012).

Knowledge of sub-PRNs within a whole-organism system can help elucidate the homeostatic costs of that system, and this is the case with osmoregulation. Osmoregulation can be energetically expensive (Bœuf et al. 2001; Ern et al. 2014) because of the rapid proliferation and maintenance of new cell types in various organs. Thus, if fish remain outside their normal salinity range, the energetic demand of osmoregulation (and related performance costs) may be elevated until external salinity returns to levels more normally experienced; however, some energetic costs may lower over time via acclimatory responses (Laiz-Carrión et al. 2004; Tseng and Hwang 2008; Brennan et al. 2016). Therefore, declines in energetic metrics like aerobic performance during salinity transitions should represent a good proxy for estimating costs of the system (Kolok and Sharkey 1997; Swanson 1998; Plaut 2001 ; McKenzie 2001 ; Velotta et al. 2018). However, evidence of increased metabolism at lower scales of biological organization (like at the cellular or tissue level via reductions of glycogen, increases in ATP consumption, or increased lactate levels) could also be informative and provide insight at the sub-PRN level (e.g., Laiz-Carrión et al. 2004). Homeostatic costs can also be incurred during salinity transitions due to hormonal pleiotropy and the ensuing tradeoffs with other organismal functions (Mauro et al. 2020). For example, cortisol, in addition to its hypo-osmoregulatory function in the gills, also affects other organismal systems. Elevated cortisol levels in fish have adverse effects on aggression/social status due to interactions with neurotransmitters in the brain and on immune function in part because of a suppression of lymphocyte proliferation (Harris and Bird 2000; Gilmour et al. 2005; Cuesta et al. 2007; Schreck and Tort 2016). Thus, investigating the immune system or behavioral performance may be helpful in quantifying the homeostatic costs of osmoregulation, for example, by quantifying declines in aggressive behaviors during competitive interactions or declines in lymphocytes during salinity transition (Cuesta et al. 2007; Mauro et al. 2025).

### The ecological consequences of the Trinidadian guppy’s hypo-osmoregulatory system

On the island of Trinidad, *Poecilia reticulata* (the Trinidadian guppy) is only found in freshwater (Torres-Dowdall et al. 2013) despite persisting in brackish water in other parts of its range (Magurran 2005; Hulsman et al. 2008) and in seawater in the lab (Chervinksi 1983; Shikano et al. 2001). In contrast, the closely related *Poecilia picta* is primarily found in brackish water on Trinidad, but it coexists with *P. reticulata* in sections of freshwater just beyond where freshwater transitions to brackish water (Torres-Dowdall et al. 2013). Lab growth experiments reveal that both species achieve their highest growth rate when food is abundant regardless of salinity. But, as food becomes more limited, growth in brackish water reduces more rapidly than growth in freshwater, and this effect is more pronounced in *P. reticulata* than in *P. picta*. This suggests that *P. reticulata* is less tolerant to higher salinity levels than *P. picta*, that the energetic cost of hypo-osmoregulation is higher in *P. reticulata* than in *P. picta*, and this in turn shapes how these fish are distributed on Trinidad (Mauro et al. 2025).

However, other homeostatic costs during hypo-osmoregulation may also contribute to *P. reticulata’s* restriction to freshwater on Trinidad. When survival was measured in *P. reticulata* in brackish water and freshwater during transplant experiments in the lab and nature, there were no differences when *P. reticulata* were alone or competing with *P. reticulata*. However, when *P. picta* competitors were present, *P. reticulata* performed much worse in brackish water than in freshwater. Additionally, behavioral experiments found that *P. reticulata* are socially subordinate to *P. picta* in brackish water but not in freshwater (Mauro et al. 2021, 2025). Collectively, these results suggest that *P. picta* out-compete *P. reticulata* in brackish water but not in freshwater due to a trade-off between hypo-osmoregulation and competitive ability; or, in other words, *P. reticulata* fitness is reduced when they attempt to maintain ion homeostasis *and* compete against *P. picta* in brackish water (Mauro et al. 2021). This trade-off appears to arise because of hormonal pleiotropy across the sub-PRNs that control osmoregulation and aggression in teleost fish (Mauro et al. 2020). A recent study used a combination of transcriptomics and behavioral measurements to find evidence for such hormonal pleiotropy in *P. reticulata*. Specifically, aggressive behaviors during a salinity transition were found to be negatively associated with increased activity of the networks (e.g., expression of receptors, binding proteins, etc) associated with the hormones predicted to be involved in the tradeoff in brain and gill tissue (e.g., cortisol, GH, insulin growth factor) (A. A. Mauro and C. K. Ghalambor, unpublished). Overall, the high homeostatic cost of hypo-osmoregulation in *P. reticulata* within the context of Trinidad’s environment (brackish water with *P. picta*) is likely why *P. reticulata* behaviorally avoid brackish water in Trinidad (Torres-Dowdall et al. 2013). Hence, the freshwater/brackish water transition area in Trinidadian may be the point at which *P. reticulata* stress shifts from the optimal state to the pejus or pessium state.

Research on *P. reticulata* on Trinidad exemplifies several important practical and ecological consequences of homeostatic systems. (1) The costs of homeostatic systems may not be apparent unless organisms are measured in natural-like settings that include multiple stressors. In *P. reticulata*, excess food in the lab masked the energetic cost of hypo-osmoregulation, and being shielded from competing with *P. picta* masked the trade-off cost of hypo-osmoregulation (Mauro et al. 2025). (2) Because environments contain multiple stressors, the sub-PRNs involved are similarly complex and may affect many homeostatic functions. Thus, a response to one stressor may influence an organism’s ability to handle additional stressors (Todgham and Stillman 2013). (3) Network studies can help dissect which types of homeostatic costs are occurring, especially in regard to the effects of multiple stressors (Schwartz and Bronikowski 2013). In *P. reticulata*, there was evidence at the phenotypic level for a negative interaction between hypo-osmoregulation and competition in brackish water (Mauro et al. 2021). This could have been purely because of the increased energetic cost of osmoregulating in brackish water. However, a network approach helped illuminate that overlapping sub-PRNs also contribute to the negative interaction (see also Kelly et al. (2016), who used a similar approach to see if overlapping sub-PRNs contributed to a negative interaction between temperature and salinity stress in a copepod). (4) Differences between the homeostatic systems of similar species can impact their ecologies. The work on *P. reticulata* and *P. picta* suggests that differences in their osmoregulatory systems have a meaningful impact on their behavior, physiology, and distributions.

### The evolution of osmoregulation in landlocked alewives

Alewives (*Alosa pseudoharengus*) are anadromous (born in freshwater and then migrate to sea as juveniles, returning as adults to freshwater to spawn), and as such, maintain a wide salinity tolerance at key life stages. For example, juvenile alewives are tolerant of freshwater and seawater (Velotta et al. 2014), as are juveniles of the congeneric *Alosa sapidissima*, at least until out-migration to sea begins (Zydlewski and McCormick 1997). As should be the case across all anadromous species—to which there are many in several taxonomic orders (Schultz and McCormick 2013)—osmotic stress leads to changes in organ and cellular level sub-PRNs that allow for the maintenance of homeostasis. Through osmosensing and PRN remodeling, anadromous fishes like alewives maintain an optimal stress response to salinity challenges to reestablish homeostasis and preserve fitness. Such a response to a change in salinity should incur a homeostatic cost (e.g., Velotta et al. 2018) that is likely transient. A unique aspect of alewives is that their ancestral osmoregulatory flexibility has evolved independently in several populations that have become permanently restricted to freshwater (hereafter landlocked). Below, we describe how the alewife system highlights the evolution of the osmotic stress response and homeostatic costs through the remodeling of osmoregulatory sub-PRNs.

Around 300–400 years ago, dams built during the colonization of New England trapped formerly anadromous populations of alewife in their natal freshwater ponds on several independent occasions (Palkovacs et al. 2008). These landlocked populations are no longer exposed to seawater in their lifetimes. This restriction to freshwater has led to the repeated degradation of a suite of hypo-osmoregulatory functions in landlocked populations (compared to the ancestral anadromous populations), including reduced Na^+^, K^+^-ATPase (ion transporter) activity and reduced transcription of ion secretory proteins in the gills (Velotta et al. 2014, 2015, 2017). This reduction of hypo-osmoregulatory flexibility in landlocked fish has led to reduced hypo-osmoregulatory performance and survival in seawater in the lab (Velotta et al. 2014), a phenomenon that worsens with age of the population since the time of establishment (Velotta et al. 2015). These data suggest that landlocked alewives suffer fitness losses in their ancestral seawater environment due to relaxed selection on the active excretion of excess Na^+^ and Cl^−^ ions gained passively from the hyper-osmotic environment.

Despite this loss of hypo-osmoregulatory function, some landlocked alewives maintain enough flexibility to survive in seawater for prolonged periods in the lab. Mortality in seawater is ubiquitous only at 40 ppt (an extra-environmental level for alewives) and only in one landlocked population (Velotta et al. 2015). In a common laboratory environment, survival of landlocked alewives in full-strength seawater (35 ppt) varies from 70 to 90%, compared to ∼99% for anadromous fish (Velotta et al. 2015). At a slightly lower salt concentration (30 ppt) survival is nearly 100% in individuals from all populations, including landlocked (Velotta et al. 2015).

Yet, despite their ability to survive, hypo-osmoregulation in landlocked alewives incurs a higher homeostatic cost compared to their anadromous counterparts. First, surviving landlocked alewives exhibit elevated blood plasma osmotic pressure compared to anadromous alewives during acclimation to 30–35 ppt seawater across a 14-day acclimation period (Velotta et al. 2015). Elevated osmotic pressure of blood plasma can lead to imbalances in intracellular fluid, which in vertebrates must be tightly regulated for proper function of proteins (McNab 2002) and thus is indicative of a homeostatic cost. Further, in a separate study, we found that juvenile landlocked alewives feed poorly at 35 ppt and subsequently lose weight over 4 weeks compared to anadromous fish, which grow in length and body condition (J. Velotta, E. Schultz, S. McCormick, unpublished). Thus, although landlocked alewives maintain the flexibility to survive in full-strength seawater for some period (days to weeks), their fitness is threatened. Overall, landlocked alewives have likely evolved reductions in hypo-osmoregulatory ability that put them between the pejus and pessium levels when in seawater. Therefore, fitness in seawater in landlocked alewives is not assured, despite maintenance of some hypo-osmoregulatory flexibility.

The evidence suggests that, in landlocked alewives, parts of the osmoregulatory sub-PRN have been remodeled or broken. For example, mRNA transcripts of genes involved in osmosensory signal transduction, gill epithelial remodeling, and regulation of gill ion channels are less responsive to seawater in landlocked fish compared to anadromous ones (Velotta et al. 2017), suggesting that fewer seawater-type ionocytes are produced in response to salt. This reduction in expression, which likely underlies the loss of hypo-osmoregulatory tolerance in landlocked fish, may be due to a remodeling of the osmoregulatory sub-PRN at any level. We hypothesize, however, that this remodeling involves the reception of hormone signals and/or the transduction of that signal to ion-exchanging cells at the tissue level (e.g., the gills). Changes to central osmosensory systems that have far-reaching effects (e.g., the osmosensing and release of cortisol from the adrenal glands) would likely have negative pleiotropic consequences that should constrain evolutionary change (Mauro et al. 2020). Though we do not yet know the precise target of relaxed selection at the sub-PRN level, it is clear that degradation of this intricate network leads to a substantial homeostatic cost for landlocked alewives.

More detailed knowledge of changes to the sub-PRN may help to elucidate the causes of homeostatic costs in landlocked alewives. For example, cessation of feeding may be a response to stress that allows landlocked alewives to minimize the extra energetic costs associated with digestion, since presumably all excess energy is being put toward maintaining osmotic balance (Jutfelt et al. 2021). In this case, we would expect changed interactions between the central nervous system, regulation of feeding behavior, and the osmotic stress response network. Because the fish PRN is multi-leveled and complex, more work is needed to understand whether there are particular parts of the system that are more likely to be remodeled and/or broken when fish lose their ability to be euryhaline. Nevertheless, landlocked alewives represent a unique system for understanding how PRNs are remodeled in response to novel ecological stressors and how that changes homeostatic costs.

## Conclusion

A grand challenge at the interface of ecology, evolution, and physiology is understanding how organisms “walk the tightrope” between stability and change in response to changing environmental conditions (Schwenk et al. 2009; Padilla and Tsukimura 2014). We hope our perspective—rooted in systems thinking—will benefit physiologists, ecologist, and evolutionary biologists as they explore how organisms respond to environmental variation. Although our examples here were focused on the homeostasis of osmolality during salinity stress in euryhaline fish, we point to small endotherms exposed to heat stress (e.g., McKechnie and Wolf 2019), animals (especially invertebrates and small vertebrates) exposed to hypoxia (e.g., Gorr et al. 2010; Cheviron et al. 2012), and insects exposed to freezing (Sinclair et al. 2003; Teets and Denlinger 2013) as systems to seek out as additional examples. Further, we suggest that the concepts presented will be applicable to other study systems if (1) the link between the environmental change and the relevant homeostatic system is clear, (2) there is some knowledge of how the sub-PRNs underlying the homeostatic response respond, (3) homeostatic costs can be linked to whole organism performance, and (4) the organism’s stress response can be placed within the context of its environment. This will be most easily achieved in studies that (1) combine fieldwork and lab work, (2) use a performance metric that can be related to fitness in a natural setting, (3) measure parts of the PRN with and without the stressor using transcriptomics, hormonal assays, or proteomics across different tissues and organs, and (4) estimate the cost of the homeostatic system under stress (see Box 2). Thus, we encourage technological developments that will make measuring these traits/metrics in non-model organisms easier (Burnett et al. 2020; Gormally and Romero 2020), analytical developments that will aid in the combinatorial analysis of different types of biological data, and the development of models that link lower-level physiological processes to population level outcomes (e.g., Kramer 2011 , Murphy 2018). Lastly, we agree with the assertion that it is important to study homeostatic systems under ecologically relevant stress levels rather than under extreme levels in which those systems break (Woods and Wilson 2015). At least in the cases of the Trinidadian guppy and alewives, it is clear that how homeostatic systems perform at lower stress levels can have ecological and evolutionary consequences.

## Data Availability

No novel data was used nor any analyses performed in this perspective paper; contact us for assistance with accessing referenced data papers if needed.

## Notes

From the symposium “Organismal Systems Biology ” presented at the annual meeting of the Society for Integrative and Comparative Biology, January 3-7th , 2025.”
